# The seroprevalence of antithyroid peroxidase antibodies in bipolar families and bipolar twins: results from two longitudinal studies

**DOI:** 10.1186/s40345-017-0070-z

**Published:** 2017-01-20

**Authors:** G. Snijders, L. de Witte, E. Mesman, S. Kemner, R. Vonk, R. Brouwer, W. A. Nolen, H. A. Drexhage, M. H. J. Hillegers

**Affiliations:** 1 Department of Psychiatry, Brain Center Rudolf Magnus, University Medical Center Utrecht, P.O. Box 85500, Heidelberglaan 100, 3508 GA Utrecht, The Netherlands; 2 Department of Psychiatry, Reinier van Arkel, Den Bosch, The Netherlands; 3 Department of Psychiatry, University Medical Center Groningen, Groningen, The Netherlands; 4 Department of Immunology, Erasmus Medical Center Rotterdam, Rotterdam, The Netherlands

**Keywords:** Thyroid peroxidase antibodies, Bipolar disorder, Offspring, Twins, Prevalence

## Abstract

**Background:**

Previous studies of our group among bipolar offspring and bipolar twins showed significant higher prevalence’s and levels of antithyroid peroxidase antibodies (TPO-Abs) in offspring and co-twins (without a mood disorder) compared to controls, suggesting that TPO-Abs might be considered as vulnerability factor (trait marker) for BD development.

**Objectives:**

Here we elucidate, in the same cohorts, but now after 12- and 6-year follow-up, whether TPO-abs should be considered as a ‘trait’ marker for BD. The present study aims to investigate whether TPO-Abs (1) are stable over time, (2) are associated with lithium-exposure, (3) share a common genetic background with BD and are related to psychopathology.

**Results:**

In bipolar offspring and twins, the prevalence of TPO-Abs is stable over time (*r*_s_ = .72 *p* < .001 resp. *r*_s_ = .82, *p* < .001) and not associated with lithium use. At follow-up, an increased prevalence of TPO-abs was again observed in bipolar offspring (10,4% versus 4%) and higher TPO-abs titers were still present in co-twins of bipolar cases compared to control twins [mean 1.06 IU/ml (SD .82) versus mean .82 IU/ml (SD .67)], although statistical significance was lost.

**Conclusions:**

Although our results show a trend toward an increased inherited risk of the co-occurrence of BD and thyroid autoimmunity, large-scale studies can only draw final conclusions. Nationwide epidemiological and GWAS studies reach such numbers and support the view of a possible common (autoimmune) etiology of severe mood disorders and chronic recurrent infections and autoimmunity, including thyroid autoimmunity.

## Background

Several cross-sectional studies demonstrated that autoantibodies to thyroid peroxidase (TPO-Abs) are about 1–2 times more prevalent in patients with bipolar disorder (BD) than in the general population (Kupka et al. 2002; Chakrabarti 2011; Bergink et al. 2013). A causative role of TPO-Abs in the pathogenesis of BD is uncertain (Bochetta et al. 2016).

TPO-Abs only play a limited causative role in the pathogenesis of autoimmune thyroid failure by aiding in the destruction of thyroid cells (Groot de et al. 2000). Autoreactive T cells specific for thyroid antigens (also part of the thyroid autoimmune response) play a more prominent role in such destruction during the inflammatory reaction in the thyroid induced by the T cell-mediated autoimmune reaction (the so-called autoimmune thyroiditis of Hashimoto) (Rosmalen et al. 2001). Nevertheless, TPO-Abs are considered clinically useful serum markers of a developing or ongoing autoimmune thyroiditis (AITD) and they often precede the clinical thyroid failure phase (i.e., raised TSH, reduced fT4) of autoimmune thyroiditis for many years. In the general population, the prevalence rate of TPO-Abs ranges from about 2–30% (Kabelitz et al. 2003), depending on age (female > men) and gender (old > young).

The thyroid autoimmune response is an aberrant response to self-antigens. Genes such as MHC-related genes, genes involved in T cell regulation (CTLA-4, PTPN22) (Tomer and Huber 2009), and environmental factors as iodine (lithium in mood disorders) play prominent roles in the pathogenesis. Lithium has a destructive capacity for thyroid cells and might play a role in modulating the thyroid autoimmune response (Wilson et al. 1991).

In two previous studies, we searched for indications of a genetic background for the relationship BD-thyroid autoimmunity. In a first study, among bipolar offspring (Dutch bipolar offspring: DBO) with a mean age of 21 years, a borderline significant (*p* = .05) higher prevalence (9%) of TPO-Abs in the DBO cohort as compared to healthy population controls of the same age (3%) was observed, the prevalence being the highest especially in the daughters, who showed a prevalence of 11% in DBO compared to 4% in healthy controls (*p* = .008). Since TPO-Ab positivity was not associated with the presence of a mood disorder, we interpreted the finding that BD and thyroid autoimmunity shared (an) inherited vulnerability factor(s) (Hillegers et al. 2007). In a second study among bipolar twins, our main finding was that co-twins of a bipolar index twin (without a mood disorder) had a higher prevalence and titer of TPO-Abs as compared to healthy control twins. In particular, monozygotic discordant twin pairs had significant higher TPO-Abs titers than dizygotic discordant twin pairs (Vonk et al. 2007). These findings of both studies strengthened us in the view that BD and thyroid autoimmunity are rooted in the same underlying genetically determined disturbance, most likely an immune disturbance, responsible for both thyroid autoimmunity and BD development. For this reason, we considered TPO-abs as a potential vulnerability factor (‘trait’ marker) for the development of BD. However, a recently published study contrasted with above-described findings of our group. Cobo and colleagues were unable to find a familial association of BD and thyroid autoimmunity and concluded that only BD-I patients showed functional thyroid abnormalities, related to lithium treatment (Cobo et al. 2015). This study determined the presence of thyroid alterations (thyroglobulin, thyroperoxidase antibodies, TSH, and free T4 levels) in 239 individuals affected with BD, 131 first-degree relatives and 108 healthy controls, and did not find significant functional thyroid abnormalities in the group of first-degree relatives. In addition, this study did not support a familial aggregation of thyroid autoantibodies positivity in pedigrees of BD-I.

We recently were able to re-investigate the DBO children and many of the bipolar twins after 6 and 12 years, respectively (resp.), to re-evaluate the prevalence of TPO-Abs (using the same techniques) in the two high-risk populations for BD. In the DBO cohort, we also assessed TPO-Abs titers in both biological parents, i.e., the bipolar index parent and the co-parent. The present study aims to elucidate whether TPO-abs is a ‘trait’ marker for BD. Therefore, we evaluated whether TPO-Abs (1) are stable over time, (2) are associated with lithium-exposure, (3) share a common genetic background (familial and twin associations) with BD and are related to the presence or development of psychopathology over time.

## Experimental procedures

### Population and procedure

The study population comprised bipolar family trios, bipolar twins, and controls from “The Dutch bipolar offspring study” and “The Dutch bipolar twin study.” Both studies received approval by the Medical Ethics Committee of UMCU. A written informed consent was obtained from all the participants. A detailed description of study design, recruitment procedure, exclusion criteria of both studies can be found in Hillegers et al. (2007), (DBO) and Vonk et al. (2007) (twins). Table 1 shows an overview of the demographic characteristics.

**Table 1:** Demographic characteristics

	Offspring study						Twin study					
Time-point	5 years follow-up^a^		12-year follow-up				Baseline			6-year follow-up		
Group	Offspring (*n* = 126)	Controls (*n* = 129)^b^	Offspring (*n* = 103)	Controls (*n* = 50)^b^	Bipolar parent (*n* = 63)	Co-parent (*n* = 58)	Bipolar index twin (*n* = 51)	Co-twin (*n* = 51)	Control twin (*n* = 70)	Bipolar index twin (*n* = 31)^d^	Co-twin (*n* = 32)^d^	Control twins (*n* = 58)^d^
Female, *n* (%)	57 (55)	103 (79)	46 (45)	28 (56)	41 (66)	29 (49)	35 (69)	33 (65)	55 (79)	23 (74)	22 (69)	41 (71)
Mean age in years (SD)	NA	NA	27.9 (2.9)	26.5 (2.5)	58.1 (4.2)	58.3 (4.2)	41.3 (10.1)	41.3 (10.1)	41.2 (9.3)	49.1 (10.5)	49.3 (11.3)	48.3 (8.1)
Range	12–21	12–19	22–32	22–33	50–68	49–67	20–60	20–60	20–57	29–69	21–69	32–72
DSM-IV diagnosis												
Bipolar disorder, n (%)	13 (10.3)		14 (13.6)	–	63 (100)	0 (.0)	51 (100)	11 (21.6)	–	21 (100)	5 (15.6)	0 (.0)
Type 1			4				38	8		24	1	
Type 2			11				13	1		7	3	
NOS								2			1	
Other			2									
Unipolar disorder, *n* (%)	37 (29.4)		42 (40.8)			8 (13.8)		8 (15.7)			5 (15.6)	3 (5.2)
Other disorder, *n* (%)^c^	23 (18.3)		19 (18.4)			4 (6.9)		4 (7.8)			9 (28.1)	8 (13.8)
No disorder, *n* (%)	53 (42.0)		28 (27.2)			46 (79.3)		28 (54.9)			13 (40.6)	47 (81.0)
Lithium use, current, *n* (%)	4 (3.9)	–	4 (3.9)	–	33 (52.4)	1 (1.7)	37 (72.5)	9 (17.6)	–	17 (54.8)	4 (12.5)	–
Thyroid medication use, current, *n* (%)	1 (.8)	–	1 (1.0)	–	17 (27.0)	4 (6.9)	7 (13.7)	3 (5.9)	2 (2.9)	7 (22.5)	3 (9.4)	2 (3.4)
TPO-Abs positivity, *n*	11 (9)	4 (3)^e^	11 (11)	2 (4)	8 (12.8)	7 (12.1)	14 (27.5)	11 (21.6)	11 (16)	10 (32.3)	7 (21.9)	8 (13.7)

^a^Results showed the maximum total of persons during the 5-year follow-up

^b^Control group in bipolar offspring is not followed longitudinally

^c^Other disorder included; psychotic disorders, anxiety disorders, substance abuse disorders, attention deficit hyperactive disorders, adjustment disorder, pervasive developmental disorder, conduct disorder

^d^Two bipolar index twins and three co-twins and four healthy control twins were from an incomplete twin pair

^e^Based on a Chi-square test, there is a statistical significant difference between offspring and controls (*p* = .05)

#### Bipolar family trios

The bipolar family trios described herein belong to the Dutch bipolar offspring study in short, 140 offspring (mean age 16 years, range 12–21) from 86 families with one parent diagnosed with BD (74% BD type I and 26% BD type II) were recruited in the years 1997–1999 and followed for 12 years. Subjects were assessed at four time-points: baseline and 1-, 5-, and 12-year follow-up with a retention rate of 77% (*n* = 108) (Mesman et al. 2013). Thyroid parameters assessments have been part of all four measurements, and (Hillegers et al. 2007) have described the results of 5-year follow-up in detail. At 12-year follow-up, at least one blood sample was available of 103 offspring subjects and 121 parents (bipolar index parent *N* = 63 and co-parent *N* = 58) from 69 bipolar family trios. The control group consisted of 50 age- and gender-matched subjects and was enrolled through medical staff via our departments.

#### Bipolar twins

Thyroid parameters have been measured at baseline and 6-year follow-up in BD and control twins. Vonk et al. (2007) have described the results at baseline. At 6-year follow-up, 53.2% of the BD twins, respectively, 45.7% of the control twins dropped out from the study. 25 twin pairs affected with BD (10 MZ discordant, 2 DZ concordant, and 13 DZ discordant) and four twins from incomplete twin pairs (two DZ co-twins from discordant pairs, two MZ bipolar index twins from discordant pairs), and 18 control twin pairs (10 MZ and 8 DZ) plus two MZ control twin from incomplete pairs were followed over time. In addition, we included 4 new enrolled twin pairs with BD (2 MZ concordant, 2 DZ discordant) and 1 twin from an incomplete pair (one MZ co-twin from concordant pair), and 9 new enrolled control twin pairs (5 MZ, 4 DZ) plus one MZ and one DZ control twin from incomplete pairs. Control twins were matched for age, gender, and zygosity.

### Psychopathology

Psychiatric status of offspring and twins at the last measurement was determined based on the SCID-I (Structured Clinical Interview for DSM-IV) (First et al. 1997). Psychiatric diagnosis of the parents was based on the International Diagnostic Checklist (Hiller et al. 1993), Family History Research Diagnostic Criteria (FH-RDC) (Andreasen et al. 1977) method, and confirmative information from the treating psychiatrist. See Table 1 for an overview of the psychopathology.

### Autoantibody- and thyroid hormone-related assessments

We refer to our previous papers for a detailed description and methodology of the thyroid parameter assessments (Vonk et al. 2007; Hillegers et al. 2007).

### Statistical analysis

Statistical analyses were performed using SPSS Statistics 22.0. The seroprevalence of TPO-abs (>25 U/ml) between various groups was analyzed as dichotomized variable (positive/negative) with a Chi-square test. Correlations were determined using Spearman’s rank correlation coefficient (*r*_s_). The absolute TPO-Abs values were non-normally distributed; a log transformation was applied. The log TPO-Abs values and covariates (age and gender) were pairwise analyzed using repeated measures analysis of covariance (ANCOVA). A detailed explanation of this analysis can be found in Vonk et al. (2007). Results were adjusted for multiple tests with the Bonferroni correction (i.e., by dividing the probability level of *p* < .05 by the number of performed tests, which resulted in a significance threshold of *p* = .002).

## Results

### Stability of TPO-Abs over time

5 DBO subjects and 5 twins (4 healthy controls and 1 BD index) developed TPO-Ab positivity in the time frames of the study (6 resp. 12 years). Switching from TPO-ab negativity to TPO-Ab positivity was not related to the presence of a BD diagnosis or other psychopathology (data not shown). Only one twin (non-bipolar co-twin) switched from positivity (239 IU/ml) during the first measurement toward negativity this time (6.66 IU/ml). An exploratory analysis using a Spearman’s rank correlation showed a positive correlation of TPO-Ab positivity over time in offspring and twins (*r*_s_ = .82 *p* < .001, resp. *r*_s_ = .72, *p* < .001). Due to the in general TPO-Ab stability over time, we investigated the relationship between TPO-Ab and psychopathology in twins and offspring at endpoint of follow-up (6 resp. 12 years).

### TPO-Abs association with lithium-exposure

Lithium-exposed subjects did not differ significantly from lithium non-exposed subjects with respect to prevalence or positive TPO-Ab titers in all samples (data not shown).

### TPO-Abs in familial and twin studies, also in relation to the diagnosis of BD

#### Bipolar family trios

Similar to our previous reports, the prevalence of TPO-ab positivity in DBO was higher (10, 4% versus 4%), yet this difference lost significance compared to previous findings at baseline (*p* = .22). However, it must be noted that 5 of the 11 TPO-Abs positive DBO cases of 2004 were lost to follow-up (Table 1). In the DBO cohort, TPO-Abs positivity was independent from the vulnerability to develop a mood disorder; of the 11 TPO-Abs positive subjects, 9.1% were bipolar, 54.5% unipolar, and 36.3% had no disorder, whereas of the 92 TPO-Abs negative subjects, 14.1% were bipolar, 39.1% unipolar, and 26,1% had no disorder and 20.7% other disorders (*p* = .33). With regard to the parents, TPO-Abs positivity was not related to BD; 12.7% of the bipolar parents (*n* = 63) compared to 12.1% of the co-parents (*n* = 58) were TPO-Abs positive (*p* = .56) (Table 1).

#### Twin cases

Bipolar subjects (*n* = 36) and their non-bipolar co-twins (*n* = 27) showed an increased prevalence compared with controls (*n* = 58) (27.7 resp. 25.9 resp. 13.8%), but differences did not reach statistical significance (*p* = .20) (Table 1). Using an ANCOVA (adjusted for age and gender) on log-transformed absolute TPO-abs values, borderline significant higher TPO-abs levels in bipolar patients (*n* = 36) as compared to controls (*n* = 58) were found (mean 1.09 IU/ml (SD .77) resp. mean .82 IU/ml (SD .67), *p* = .076), the log-transformed TPO-abs values of the non-bipolar co-twins (*n* = 27) were between those of the bipolar patients and control twins (mean 1.06 IU/ml (SD .82, *p* = .83 resp. *p* = .23). In the previous study of Vonk et al. (2007), differences in mean levels of absolute TPO-Abs values (using a logarithmic transformation) reached significance, i.e., bipolar patients had higher mean TPO-Abs levels compared to control twin pairs (*p* = .02). A pairwise repeated measures ANCOVA on the log-transformed absolute TPO-Abs values showed higher levels in the discordant BD twin pairs compared to control pairs (mean 1.11 IU/ml (SE .11) mean .84 IU/ml (SE .12), *p* = .12), particularly in MZ discordant twin pairs. Differences were not found in mean log-transformed TPO-Abs levels between BD index and non-bipolar co-twins. All these findings are consistent with previous results (Vonk et al. 2007), yet were now not significant (probably due to the smaller sample size).

## Discussion

We here show that the prevalence and titer of TPO-Abs is bye-and-large stable over a time span of 6–12 years in two cohorts. This is in accord with the view that there is a bye-and-large stability in the prevalence of TPO-Abs in age groups of 20–60 years of age (females 10–20%, males 2–8%) with a sharp increase in prevalence of TPO-Abs positivity during aging in men over 60 years of age (Bjoro et al. 2000; Rosmalen et al. 2001). These findings indicate that TPO-abs positivity should be considered as a ‘trait’ marker, rather than being related to mood state.

Similar to previous reports, increased rates of sero-positivity and levels of TPO-abs in relatives (offspring or co-twin) of bipolar index cases as compared to controls were observed, irrespective of the presence of a mood disorder. However, in contrast to earlier studies, the results were not significant; while in the previous series of 2004, just significant values of *p* = .05 and *p* < .05 were found in DBO and BD twins respectively and in the second series of 2010, these significant values were absent (*p* = .22. and *p* = .12). We assume that the significance discrepancy between the initial and follow-up studies can be explained by the power limitations of the studies and the fact that 5 of the 11 TPO-abs-positive DBO cases of 2004 were lost at follow-up. Assuming that TPO-Abs positivity is stable during the follow-up (see results), the prevalence of TPO-Abs positivity remains significantly increased compared to controls (*p* = .04), when taking into account earlier data of the 5 drop-outs at follow-up.

For the twins, >45% were lost at follow-up compared to the previous measurement, even though a few new pairs were enrolled and included in the analysis, the sample size was still small (*N* = 121). Our findings of borderline (non-) significant differences in the follow-up of both cohorts are congruent with the mixture of positive and negative reports (Bartalena et al. 1990; Kupka et al. 2002; Radhakrishnan et al. 2013; Haggerty et al. 1997; Cobo et al. 2015; Vonk et al. 2007; Oomen et al. 1996) on a genetic association of BD and thyroid autoimmunity and our inability to detect a higher prevalence of TPO-Abs in the bipolar parent of the DBO study. The major drawback of these conflicting studies on the familial relationship between major mood disorders and thyroid autoimmunity is often the sample size. Large numbers have been obtained in nationwide epidemiological studies which have—with the insight of today—a more advanced and sensible set-up given the limitations of the small-scale cohort studies. A recent Danish nationwide study reported that autoimmune diseases are risk factors for subsequent mood disorders; the incidence rate ratio (IRR) for autoimmune thyroiditis was 1.05 (.72–1.52 95% CI) in mood disorder patients without chronic infections and 1.63 (1.09–2.23 95% CI) for mood disorder patient with chronic infections (Benros et al. 2013). The IRR was even higher for Graves’ disease, namely 1.28 (1.12–1.45 CI) in mood disorder patients without infections and 1.90 (1.63–2.21 CI) for mood disorder patients with infections. In general, a prior hospital contact because of autoimmune disease or any history of hospitalization for infection increased the risk for later mood disorder diagnosis by 45% (IRR, 1.45; 95% CI 1.39–1.52) resp. 62% (IRR, 1.62; 95% CI 1.60–1.64) (Benros et al. 2013). GWAS studies in psychiatric and autoimmune patients support the view of shared genetically determined abnormal immune interactions (The Network and Pathway Analysis Subgroup of the Psychiatric Genomics Consortium 2015). These associations are compatible with the immune hypothesis for the development of mood disorders in at least a subgroup of mood disorder patients. However, we are not able to identify this subgroup of mood disorder patients since our studies do not find a direct relationship between psychopathology and the presence of TPO-Abs. Hence, future research should be directed toward investigating TPO-Abs in larger BD samples, also in relation to clinical features. Based on our results, we do not recommend screening for TPO-Abs in BD patients and first-degree relatives, as a marker for mood disorder vulnerability. Although in the presence of thyroid-related symptoms or aberrant thyroid hormone levels, determination of TPO-abs might be important as part of the clinical diagnostic work-up.

Collectively, our previous and present and particularly data from epidemiological and GWAS studies support the view that there is an increased inherited risk of co-occurrence of severe mood disorders and chronic recurrent infections and autoimmunity, including thyroid autoimmunity, the latter only with a limited OR of between 1 and 2. This means that only large-scale studies will unveil the association mood disorder-thyroid autoimmune disease as measured by the prevalence of TPO-Abs.

## Abbreviations

TPO-Abs: antithyroid peroxidase antibodies
AITD: autoimmune thyroiditis
BD: bipolar disorder
DZ: dizygotic
DBO: Dutch bipolar offspring
FH-RDC: family history research diagnostic criteria
IRR: incidence rate ratio
MZ: monozygotic
MD: mood disorders
resp.: respectively
SCID-I: Structured Clinical Interview for DSM-IV
SD: standard deviation
